# The effects of an extensive exercise programme on the progression of Mild Cognitive Impairment (MCI): study protocol for a randomised controlled trial

**DOI:** 10.1186/s12877-017-0457-9

**Published:** 2017-03-22

**Authors:** Kate E. Devenney, Brian Lawlor, Marcel G. M. Olde Rikkert, Stefan Schneider, on behalf of the NeuroExercise Study Group, Justine A. Aaronson, Vera Abeln, Jurgen A. H. R. Classen, Robert F. Coen, Emer M. Guinan, Damien Ferguson, Roy P.C. Kessels, Romain Meeusen, Christian Montag, Ross T. Murphy, M. Cristina Polidori, Martin Reuter, Marit L. Sanders, Heiko K. Strüder, Dick H.J. Thijssen, Tobias Vogt, Cathal Walsh, Bernd Weber, Jennifer Hoblyn, Andrew Eustace, Cora McGreevy, Aisling Denihan, Justin Kinsella, Declan Lyons, Sean Kennelly

**Affiliations:** 1 Discipline of Physiotherapy, Trinity College Dublin, Dublin, Ireland; 2 Trinity College Institute of Neuroscience, Dublin, Ireland; 3 Department of Geriatric Medicine, Radboud Alzheimer Centre, Radboud University Medical Center, Nijmegen, The Netherlands; 4 Institute of Movement and Neurosciences, German Sport University Cologne, Cologne, Germany; 5 Faculty for Science, Health, Education and Engineering, University of the Sunshine Coast, Maroochydore, Australia

**Keywords:** Mild cognitive impairment, Exercise intervention, Physical activity, Cognitive function, Brain structure, Frailty, Epigenetics

## Abstract

**Background:**

Exercise interventions to prevent dementia and delay cognitive decline have gained considerable attention in recent years. Human and animal studies have demonstrated that regular physical activity targets brain function by increasing cognitive reserve. There is also evidence of structural changes caused by exercise in preventing or delaying the genesis of neurodegeneration. Although initial studies indicate enhanced cognitive performance in patients with mild cognitive impairment (MCI) following an exercise intervention, little is known about the effect of an extensive, controlled and regular exercise regimen on the neuropathology of patients with MCI. This study aims to determine the effects of an extensive exercise programme on the progression of MCI.

**Methods/design:**

This randomised controlled clinical intervention study will take place across three European sites. Seventy-five previously sedentary patients with a clinical diagnosis of MCI will be recruited at each site. Participants will be randomised to one of three groups. One group will receive a standardised 1-year extensive aerobic exercise intervention (3 units of 45 min/week). The second group will complete stretching and toning (non-aerobic) exercise (3 units of 45 min/week) and the third group will act as the control group. Change in all outcomes will be measured at baseline (T0), after six months (T1) and after 12 months (T2). The primary outcome, cognitive performance, will be determined by a neuropsychological test battery (CogState battery, Trail Making Test and Verbal fluency). Secondary outcomes include Montreal Cognitive Assessment (MoCA), cardiovascular fitness, physical activity, structural changes of the brain, quality of life measures and measures of frailty. Furthermore, outcome variables will be related to genetic variations on genes related to neurogenesis and epigenetic changes in these genes caused by the exercise intervention programme.

**Discussion:**

The results will add new insights into the prevailing notion that exercise may slow the rate of cognitive decline in MCI.

**Trial registration:**

ClinicalTrials.gov NCT02913053

## Background

With an advancing aging population and associated rise in dementia prevalence in developed countries, the associated costs and disease burden have exerted significant pressure on economic and social systems [1]. To help ensure an aging society live enjoyable and productive lives, research into treating or preventing conditions such as Alzheimer’s disease (AD) and other forms of age-related neurodegenerative diseases is an urgent public health priority. Today, longevity-related prevalence of neurodegenerative diseases and especially dementia, along with the current absence of a cure are among the top prominent societal health-related challenges acknowledged by the first G8 Summit on dementia held in London on December 11th, 2013 [2].

Published diagnostic criteria for AD in Lancet Neurology diagnose Mild Cognitive Impairment (MCI) as the preclinical or prodromal stage of AD [3]. In MCI, several of the clinical and neuropsychologic pathologic features are present prior to the onset of overt AD [4]. Patients with MCI in the earliest stage of neurodegeneration can be clinically diagnosed, and represent a patient cohort consistently able to participate in a structured exercise programme. Consequently, there has been an increased research focus on both pharmacological and non-pharmacological strategies to optimise cognitive function and enhance ‘brain health’ in older age [5], particularly for individuals at risk of developing AD [6].

Previous cross-sectional studies have established that moderate activity during midlife is associated with a lower risk of having MCI in later life, with late-life participation in moderate exercise also associated with lower risk for MCI [7]. A meta-analysis of randomised controlled trials (RCTs) by Heyn et al. [8] reported beneficial effects of physical activity on physical fitness and cognitive function in adults with cognitive impairment. In recent years, a broad range of exercise intervention studies have demonstrated cognitive benefits can be achieved with varying exercise modalities in populations with MCI [9–11]. Aerobic exercise has demonstrated significant improvements in global cognitive scores with a weak but significant effect on memory [12]. A meta-analysis by Gates et al. [13] examining the effects of chronic exercise training on cognitive function in older adults with MCI found research quality was modest, with many studies under-powered and only 8% of cognitive outcomes demonstrating statistically significant change. A limitation across a number of these studies is the small sample size and variation in MCI diagnostic criteria applied. The differing exercise approaches used across exercise intervention studies, coupled with the wide variation in cognitive tasks utilised make it difficult to summarise and synthesise research findings [14].

There is converging evidence from animal and human studies that regular aerobic exercise acts as a promoter of ’brain health‘ mediating neural homeostasis and, via neuroprotective and neurorestorative mechanisms, thereby counteracting brain ageing. At the behavioural level, exercise has been found to upregulate affective states [15, 16] and to improve cognition throughout different age phases [17–19] and different dimensions, including spatial/associative learning [20, 21], attentional processing [22], and executive control [23]. While animal research has allowed the unravelling of the underlying neurobiological mechanisms of exercise at the behavioural (e.g. water maze-type tests), cellular (e.g. neurogenesis, synaptogenesis, neuroangiogenesis), and humoral (e.g. neurotrophic factors, inflammatory cytokines) levels [24, 25], so far the neurobiological and epigenetic effects of exercise remains poorly understood in humans. While preliminary results indicate that aerobic exercise inhibits the progression of AD-like neuropathology in an animal model [26, 27], currently there is little information about the effects of regular physical exercise on the progression of both functional and structural markers at the pre-dementia and early dementia stages in humans.

The aim of this study is to compare a 12-month structured exercise programme (aerobic and stretching and toning group) to a control group for progression of cognitive decline in MCI. The stretching and toning group will act as a non-aerobic exercise group, controlling for the social effect of a structured group exercise programme. This type of low-intensity exercise intervention has previously shown some positive effects on cognitive outcomes, as it provides participants with equivalent opportunity for social interaction [28]. Previous exercise intervention studies in MCI that have utilised non-aerobic exercise (e.g. stretching and toning) as a control intervention have noted some improvement in cognitive performance [8, 29] while others have not demonstrated significant change in cognitive performance [9, 10]. The comparison between aerobic exercise, non-aerobic exercise and a control group coupled with the 12-month intervention period, concomitant brain scanning, genetic and epigenetic analyses is innovative and will be a strong addition to the growing body of literature around exercise in MCI.

## Methods/design

### Study aims

The primary aim of this study is to investigate the effects a 12-month structured exercise programme (aerobic, and stretching and toning group) compared to a control group for the progression of cognitive decline in MCI. The primary hypothesis of this study is that participation in an extensive exercise programme will demonstrate a slower rate of cognitive decline compared to the control group. A secondary hypothesis is that participants in the aerobic exercise group will demonstrate a stronger positive effect of cognitive functioning than the stretching and toning (non-aerobic) group.

The secondary aims of this study are:

1. To examine the effects of a structured exercise programme on MoCA scores, a screening tool of global cognition in MCI
2. To determine the effects of a structured exercise programme on cardiovascular fitness
3. To measure the effects of a structured exercise programme on physical activity levels
4. To investigate a mechanism of action through epigenetic analysis and exploration of structural and functional changes on Magnetic Resonance Imaging (MRI) brain pre and post 12-month intervention
5. To investigate the effects of a structured exercise programme on quality of life measures and measures of frailty

### Study design

**Fig. 1:**
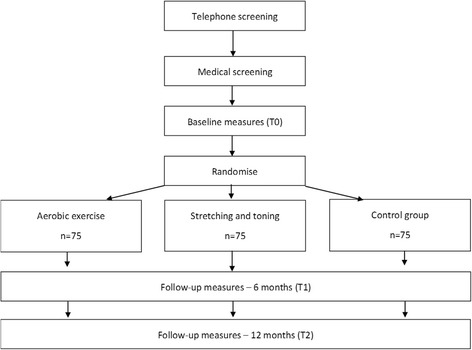
Participant flow through study

This proof of concept will take the form of a randomised controlled trial to be completed across three centres in Europe; the German Sport University Cologne, Germany, University of Nijmegen, The Netherlands and Trinity College Dublin, Ireland. A total of 225 participants will be randomised (*n* = 75 at each site) to either a yearlong supervised and home based aerobic exercise programme (*n* = 75), an equivalent non-aerobic stretching/toning programme (*n* = 75) or to the control group (*n* = 75). The primary outcome will be change in cognitive performance as measured by a neuropsychological test battery. Secondary outcomes will include changes in MoCA scores, cardiovascular fitness, physical activity, quality of life measures, measures of frailty, epigenetic and structural changes. Change in all outcomes will be measured at baseline (T0), after six months (T1) and after 12 months (T2) (see Fig. 1).

### Recruitment and screening

In total, 225 previously sedentary adults aged 50 years or older who are diagnosed with MCI will be recruited via hospital memory clinics affiliated with the three sites and from the community. Advertising will take place through community centres and newspaper articles. Initial screening will be completed over the telephone to determine eligibility. Participants who meet the following diagnostic, inclusion and exclusion criteria and successfully complete baseline measures including a screening exercise test will be enrolled.

### Diagnostic criteria

Participants will have a diagnosis of MCI due to AD according to the Albert et al. [3] criteria. All enrolled participants with MCI will be classified as having memory decline but not dementia (Clinical Dementia Rating global score = 0.5), consistent with established MCI classification [3, 30].

### Inclusion criteria

Participants who meet the following criteria will be eligible to participate: (1) MoCA [31] 18–26; (2) stable medical condition for more than 6 months; (3) stable medication for more than 3 months; (4) adequate visual and auditory acuity to complete neuropsychological testing; (5) electrocardiogram without significant abnormalities that might interfere with the study; (6) physical ability sufficient to allow performance of endurance exercise training; (7) capacity to provide written and dated informed consent form; (8) medical clearance to undergo a symptom-limited cardiopulmonary exercise test and extensive aerobic exercise training.

Participants recruited from the community via newspaper articles and community advertisement will complete additional testing to determine MCI status. To distinguish between amnestic and non-amnestic MCI, agreed education adjusted cut-offs of -2 Standard Deviation (SD) for low education (<10 years of education), -1.5 SD for the middle group (10–13 years of education) and -1 SD for the highly educated (>13 years of education) will be taken from the delayed recall portion of an age adjusted episodic memory test. In Nijmegen and Dublin this will be evaluated using the Logical Memory (story recall) subtest of the Wechsler Memory Scale (WMS-IV) [32, 33]. In Cologne, education scores will be examined using the Repeatable Battery for the Assessment of Neuropsychological Status (RBANS) [34] Delayed Memory Index (Score of < 85).

### Exclusion criteria

Participants will be deemed ineligible if they meet any of the following criteria: (1) diagnosis of AD or other type of dementia; (2) history of familial early-onset dementia; (3) enrollment in any investigational drug study; (4) history in the past 2 years of epileptic seizures (participants with epilepsy who have been stable off medication or seizure free for 2 years may be included); (5) any major psychiatric disorder (a clinical diagnosis of major depressive disorder, bipolar or schizophrenia); (6) past history or MRI evidence of brain damage, including significant trauma, stroke, hydrocephalus, mental retardation, or serious neurological disorder; (7) carotid stent or severe stenosis; (8) history of myocardial infarction within previous year; (9) congestive heart failure (New York Heart Association Class II, III or IV) (10) uncontrolled hypertension or hypotension (systolic blood pressure >200 mm Hg and/or diastolic blood pressure >110 mm Hg at rest) [35]; (11) unstable cardiac, renal, lung, liver, or other severe chronic disease; (12) type 2 diabetes mellitus with hypoglycemia in the last 3 months; (13) significant history of alcoholism or drug abuse within last 10 years; (14) engagement in moderate-intensity aerobic exercise training for more than 30 min, 3 times per week, during past 2 years; (15) history of vitamin B12 deficiency or hypothyroidism (stable treatment for at least 3 months is allowed); (16) serious or non-healing wound, ulcer, or bone fracture

In Cologne and Nijmegen, participants will be invited to complete brain MRI scans. Participants with pacemakers or other medical metal devices will not be eligible for MRI scanning as per standard procedures.

### Withdrawal of participants

The investigator can decide to withdraw a subject from the study for urgent medical reasons. Subjects can leave the study at any time for any reason if they wish to do so without any consequences. All primary analyses will be performed on an intention-to-treat basis with all randomised participants included in the primary analyses. Participants who withdraw from the study will be invited to attend T1 and T2 assessments.

### Randomisation, allocation, concealment and blinding

Following baseline assessment, participants will be randomised to one of three arms using a centrally controlled computer generated randomisation list (for each country) generated by an independent statistician. Participants will be randomised to one of three arms as per Fig. 1. At each centre, the investigators will be blinded to allocation order and the treatment will be assigned using sealed envelopes based on order of recruitment. Outcome assessors and exercise trainers will not be blinded to the allocated treatment arm.

## Interventions

### Exercise intervention

The aim of both the aerobic intervention and stretching and toning exercise intervention groups will be to accrue 3 x 45 min exercise sessions per week over 12 months. Participants will complete a combination of supervised instructor led classes and unsupervised home exercise sessions. Class attendance and adherence to unsupervised home exercise sessions will be recorded for each participant by the class instructor each week over the 12 month intervention period.

The goal of the aerobic exercise class will be to accumulate at least 45 min of extensive aerobic exercise, prescribed by heart rate (HR) calculated as 180 bpm – age. Exercise intensity will be monitored during the supervised classes using a HR monitor and subjective reporting of the exercise intensity using the Borg’s Rating of Perceived Exertion (RPE) [36]. Participants in the aerobic exercise group will aim to achieve a target RPE of 13 while exercising. Each supervised class will be comprised of a 5–10 min warm up, 45 min of targeted aerobic exercise and a 5–10 min cool down. A range of aerobic exercise modalities will be offered including cycling, treadmill walking, elliptical training, endurance related indoor activities, outdoor walking, jogging and aqua jogging.

The aim of the stretching and toning group will be to complete non-aerobic activities. Each supervised class will be comprised of a 5–10 min warm up, 45 min of stretching, balance, coordination, relaxation, group games and light resistance exercises and a 5–10 min cool down. During the stretching and toning class, exercise intensity will not exceed an RPE of 10. Participants in the stretching and toning group will not be advised about aerobic activity and will not be instructed to avoid completing routine aerobic activity. The stretching and toning group will act as a social control group and it is not anticipated to see significant improvement in study outcomes.

The control group will receive usual care and will not be advised about exercise or attend supervised sessions. Participants in the control group will complete outcome assessments.

### Outcome assessment

All outcomes will be measured at T0 (Baseline), T1 (6 months) and T2 (12 months) time points. Brain MRI, blood sampling for epigenetic analysis and the NEO-Five Factor Inventory (NEO-FFI) will only be measured at T0 and T2. All participants will undergo described tests, except for MRI (only in Cologne and Nijmegen).

## Primary outcome

### Cognitive function

The primary outcome is cognitive performance. Cognitive performance will be assessed by a neuropsychological test battery measuring six cognitive domains. The test battery will consist of a computer based CogState Battery including the International Shopping List Task (ISLT) – immediate and delayed recall, Detection Task, Identification Task, One Back Task and One Card Learning Task (https://cogstate.com/) [37, 38], Verbal fluency [39, 40] and Trail Making Test (TMT) [41].

The allocation of to the tests to the six cognitive domains is based on the CogState Guidelines and conventional classification of neuropsychological tests [42]. *Verbal memory* will be assessed by ISLT. *Psychomotor function* will be measured with the Detection Task. *Executive function* will include TMT-B, Letter Fluency and Category Fluency. *Attention* will be assessed by the Identification Task and TMT-A. *W*or*king memory* will be measured by One Back Task, and *Visual memory* by the One Card Learning Task. A description of the tasks is described below.

The CogState battery will take approximately 30 min to complete. The ISLT is a 12 word, four trial (three learning trials and one delayed recall trial). Total number of correct responses made in remembering the list on three consecutive trials at a single session and after a delay will be recorded. The ISLT has been shown to have good sensitivity to verbal memory impairment [43]. The Detection Task measures psychomotor functioning and speed of processing. Participants must respond as quickly as possible when the card shown face down in the centre of the screen flips over by pressing a button on the keyboard. Reaction time is measured with lower scores indicating better performance. The Identification Task measures visual attention. Participants must decide whether a playing card presented on screen is red, by pressing the ‘Yes’ or ‘No’ button. Reaction time is measured and lower scores indicate better performance. The One Back Task assesses working memory. Participants are presented with a sequence of playing cards in the centre of a screen and must decide if the card presented is the same as the one shown immediately before. The One Card Learning Task measures visual learning and memory. Participants are presented with a succession of playing card on screen, and must decide if the card currently displayed has been displayed previously. Accuracy of performance is measured, with higher scores indicating better performance. A number of studies have found that the CogState battery of tests are sensitive to detecting cognitive impairment in mild to moderate AD and amnestic MCI populations relative to healthy matched controls [44]. CogState and has been validated across a broad range of cognitively impaired populations [38].

Verbal fluency will be assessed with Letter Fluency [39] and Category Fluency [40]. For the Letter Fluency test participants are allowed one minute to generate as many words as possible that begin with a specific letter. This task will be repeated three times with three different letters (e.g. F, A, S). For the category fluency test, participants must give as many examples of animals as possible within one minute.

TMT will be completed as a paper and pen based task. The TMT consists of two sub trials. TMT-A require individuals to sequentially connect 25 encircled numbers on a sheet of paper, while TMT-B require participants to draw a line, alternating between numbers and letters in ascending order.

### Secondary outcomes

Secondary outcomes will include global cognitive function, cardiovascular fitness, physical activity, quality of life, depression, measures of frailty and epigenetic changes.

MoCA screening tool will be used as a broad measure of global cognitive function. The MoCA is a one-page 30-point test administered in 10 min which consists of 13 tasks covering the following eight cognitive domains: visuospatial/executive functions, naming, verbal memory registration and learning, attention, abstraction, delayed verbal memory, and orientation. It has demonstrated high sensitivity and specificity as a cognitive screening instrument and has been validated to detect MCI [31].

Cardiovascular fitness will be assessed using an incremental exercise test on a standard cycle ergometer. Participants at the German Sports University and Trinity College Dublin will complete a maximal test in accordance with the World Health Organisation Protocol [45]. The test will commence with 3 min cycling unloaded, followed by the incremental phase of exercise during which the load will increase by 25 W every two minutes until the test is terminated. Blood lactate levels and participants reported BORG RPE scores will be measured at each stage of the test (2 min intervals). At the University of Nijmegen, aerobic fitness will be estimated from a submaximal exercise test completed according to the Astrand-Rhyming submaximal protocol [46]. During the first two minutes, resistance of the ergometer will be increased until a steady state HR of 70% of the estimated maximal HR is reached. Participants continue pedalling for 6 min. HR and RPE will be recorded every minute. VO_2_max will be estimated using the average HR of minute 5 and 6 and the work load in the Astrand Nomogram.

Physical activity will be assessed objectively using an activity accelerometer to be worn for seven consecutive days by study participants as each assessment time point and subjectively with the LASA Physical Activity Questionnaire ﻿(LAPAQ). The LAPAQ questionnaire is a valid and reliable self-reported questionnaire that captures physical activity over the preceding 14 days [47].

Health related quality of life will be evaluated using the Health Related Quality of Life for People with Dementia (DemQOL). DemQOL is a 28 item interview administered questionnaire relating to different aspect of QOL. The DemQOL has been validated in a large sample of people with dementia and demonstrates good acceptability and internal consistency [48]. It has also been used in older adults and in patients with MCI [49]. In addition, the Center for Epidemiologic Studies Depression (CES-D) questionnaire will be administered to determine depressive symptoms. The CES-D Scale is a short self-report questionnaire that measures symptomatic depression [50] that has been validated as a depression screening tool in older adults [51]. Depressive symptoms are associated with increased risk of MCI [52]. The association of depression with prevalent MCI and with progression from MCI to dementia, but not with incident MCI, suggests that while depression is prevalent in MCI, it does not precede it [53].

Measures of frailty will include The Timed Up and Go (TUG) test, hand grip strength and 30 s chair stand. The TUG will assess the participant’s mobility and balance. The TUG is a reliable and valid test for quantifying functional mobility and is useful in following clinical change in frailty over time [54]. Hand grip strength will be measured using a Jamer Digital Dynamometer as a measure of upper limb strength. A standardised approach will be taken to obtaining the measurement [55]. Hand‐Grip strength has been shown to predict future outcomes in aging adults including mortality and future levels of disability [56]. The 30 s chair stand will determine lower limb strength and endurance. Lower body strength is considered critical in evaluating the functional performance of older adults [57].

Venous blood samples will be collected for genetic and epigenetics analysis performed on a Sequenom Massarray Analyzer 4 at the Department of Psychology University of Bonn, Germany. Genotyping of the Apolipoprotein E (APOE) and the Brain Derived Neurotrophic Factor (BDNF) genes and their epigenetic methylation patterns will be the primary focus. The genetic analyses will serve as predisposed risk and resilience factors for cognitive functioning and decline in MCI participants. The epigenetic findings will shed new light on the link between exercise and gene activation of relevant genes in the biochemical pathways underlying cognitive decline. Given the moderating effect of the common genetic variation of APOE via personality on AD onset [58], the NEO-Five Factor Inventory (NEO-FFI) will be included to assess personality traits such as neuroticism or extraversion, predicting an earlier onset of Alzheimer and cognitive decline in elderly humans [59]. NEO-FFI is a psychological personality inventory consisting of 60 items to measure five personality traits. The questionnaire will be completed following each blood draw to form part of the epigenetics analysis. The NEO-FFI is also discussed as a measure of emotional intelligence [60].

Structure and function of related brain regions will be measured using functional MRI (fMRI) brain which is a non-invasive method for examining brain activity and structure. Structural imaging will include isotropic T1-weighted-, T2-weighted- and FLAIR- sequences with an isotropic spatial resolution of 1x1x1mm. The combination of the different image weightings allows for an automatic detection/volumetry of white matter lesions. Additionally, the combination of these sequences improves image segmentation. The structural protocol will be rounded up by a 3D-DTI sequence (60 diffusion directions, 1.7x1.7x1.7 mm) and a resting state fMRI Table 1.

**Table 1:** SPIRIT diagram outlining schedule of enrolment, interventions, and assessments for study participants

Timepoints		Pre-screening telephone call	Baseline assessment T0	Study assessment T1	Study assessment T2
			<13 days following screening	6 months after study visit 1	12 months after study visit 1
Enrolment	Explain study	X			
	Screen eligibility criteria	X			
Study Outcomes	Neuropsychological testing		X	X	X
	MoCA		X	X	X
	DemQOL		X	X	X
	CES-D		X	X	X
	Incremental Exercise Test		X	X	X
	Physical Activity Tracker		X	X	X
	LAPAQ		X	X	X
	Blood sampling		X		X
	NEO-FFI		X		X
	MRI Brain^a^		X		X
	TUG		X	X	X
	Hand Grip Strength		X	X	X
	30 second chair stand		X	X	X
	Randomisation (after completion of all baseline assessments)		X		
	Exercise intervention^b^		X	X	X

^a^MRI brain will be performed in Cologne and Nijmegen sites

^b^After completion of T0 assessment, participants will be randomised to one of the three groups (aerobic exercise, stretching and toning exercise, control group). Exercise intervention will be continuous for a 12-month duration following completion of T0 assessments

### Safety

All serious adverse events (SAE)/adverse events (AE) will be recorded on study specific adverse event forms. All AE’s will be registered with the local principle investigator (PI). These will be discussed at regular team meetings and collected and registered at the end of the study. All SAE’s will be registered centrally. In the case of an SAE, all site PI’s will be informed both at time of occurrence (with 24 h) and for a final conclusion on causality.

### Sample size

The sample size estimation was performed in “G*Power”, a statistical software program. The effect size was estimated, based on the effect size found in several studies examining the effect of exercise on cognition in elderly with MCI or increased risk for AD [8–10, 61, 62]. Sample size was calculated based on a two-tail statistical t-test set 1-β = 0.80, α = 0.05, an effect size of 0.4, an allocation ratio N2/N1 of 2. A total sample size of 224 was calculated. Considering a correlation of 0.5 between the outcome measures at T0 and T2, the design design-factor D = 1-0.52 = 0.75. The expected dropout rate is 25%. 224*0.75*1.25 = 210. Considering the fact that the primary analysis will be a combination of the results of three different centres, the final sample size *n* = 210 is rounded up to *n* = 225.

### Statistical analysis

The primary analysis of this study will be comparison of cognitive functioning (primary outcome measure) between all three intervention arms before (T0) and after (T2) the 1 year intervention. A composite score will be calculated by averaging all six domain scores into one overall cognition score. The obtained scores per test will be converted into z-scores based on the standard deviation and mean of the total sample at baseline. In case of multiple tests within one domain, the average z-score for the domain will be calculated. Secondary outcome measures are the six separate cognitive domain scores and the other parameters.

For the primary analysis we will have an intervention group of *n* = 150 (both exercise forms together) and a control group of *n* = 75 as input of an ANCOVA with dependent variable the change in cognitive composite score between T2 and T0, and as covariates baseline cognitive functioning, sex, age. In a secondary analysis the comparison between the two exercise forms will be carried out in a similar ANCOVA analysis.

Analyses for all secondary outcome parameters will be carried out with similar ANCOVA analyses. Secondary analyses will also elucidate the contrasts between T0 and T1 and T2 for the primary and secondary outcomes, and thus elucidate the course over time, without correction for multiple comparisons, as these analyses are exploratory.

Furthermore, to assess change in physical fitness, quality of life, cerebral structure and epigenetics a similar statistical approach will be used as for the primary study parameter. A *p*-value of < 0.05 will be used to assess statistical significance.

### Data management

All data will be managed using unique study codes to protect participant confidentiality, which will be used to code and file all electronic information. The key linking this code to participant identity will be stored in a secured file, access to this key is available only to designated members of the research team at each site. Raw data will be stored in a file cabinet with a lock where only designated research team members will have access to the key.

## Discussion

To the best of our knowledge, there have been no intervention trials evaluating the effect of an extensive, controlled and structured 12-month exercise programme on the progression of cognitive decline in an MCI population. Since the neuropathological change process can take years after onset of MCI, the addition of longer intervention period may result in larger intervention effects. An important consideration of this study is the isolated aerobic exercise intervention. A number of exercise intervention studies have implemented multimodal exercise interventions [11, 63], making it difficult to interpret the effect of isolated exercise modalities. The large sample size, longer duration of exercise intervention, comprehensive neuropsychological test battery will enhance the existing research around exercise and cognitive function in MCI.

The secondary outcomes will examine several potential underlying mechanisms that may influence the exercise-cognitive relationship in MCI. The effect of exercise on brain structure and function measured by MRI brain will be examined. Methylation analyses of the APOE gene and neurotrophic genes will explore the effects of exercise on this known AD risk factor. Static gene polymorphisms will be used to predict intervention outcomes. Finally, cardiovascular fitness will be measured and examined as a moderator of the exercise-cognitive relationship in MCI. While cognitive performance is the primary outcome, we will also assess whether participation in a structured exercise programme or changes in cognition can influence quality of life and measures of frailty, known risk factors for cognitive further decline [64].

## Abbreviations

AD: Alzheimer’s disease
AE: Adverse event
ANCOVA: Analysis of covariance
APOE: Apolipoprotein E
BDNF: Brain derived neurotrophic factor
CES-D: Center for epidemiologic studies depression
DemQOL: Health related quality of life for people with dementia
fMRI: functional magnetic resonance imaging
HR: Heart rate
ISLT: International shopping list task
LAPAQ: LASA physical activity questionnaire
MCI: Mild cognitive impairment
MRI: Magnetic resonance imaging
NEO-FFI: NEO-five factor inventory
RBANS: Repeatable battery for the assessment of neuropsychological status
RCT: Randomised controlled trial
RPE: Rate of perceived exertion
SAE: Serious adverse event
SD: Standard deviation
TMT: Trail making test
TUG: Timed up and go
WMS: Wechsler memory scale
