# Erratum to: The effects of an extensive exercise programme on the progression of mild cognitive impairment (MCI): study protocol for a randomised controlled trial

**DOI:** 10.1186/s12877-017-0500-x

**Published:** 2017-05-19

**Authors:** Kate E. Devenney, Marit L. Sanders, Brian Lawlor, Marcel G. M. Olde Rikkert, Stefan Schneider, on behalf of the NeuroExercise Study Group

**Affiliations:** 1 Discipline of Physiotherapy, Trinity College Dublin, Dublin, Ireland; 2 Trinity College Institute of Neuroscience, Dublin, Ireland; 3 Department of Geriatric Medicine, Radboud Alzheimer Centre, Radboud University Medical Center, Nijmegen, The Netherlands; 4 Institute of Movement and Neurosciences, German Sport University Cologne, Cologne, Germany; 5 Faculty for Science, Health, Education and Engineering, University of the Sunshine Coast, Maroochydore, Australia

## Erratum

After the publication of this work [1] it was noticed that second author ‘Marit L. Sanders’ was incorrectly removed from the author list as a named author and listed under the NeuroExercise study group. The original article has been revised to correct this.

The Acknowledgments have been revised accordingly and should appear as below:

## Acknowledgments

Members of the NeuroExercise Study Group (in alphabetical order by name). Dr. Justine A. Aaronson, Department of Medical Psychology, Radboud University Medical Center, Nijmegen, The Netherlands; Dr. Vera Abeln, Institute of Movement and Neurosciences, German Sport University Cologne, Germany; Dr. Jurgen A.H.R Claassen, Department of Geriatric Medicine, Radboud University Medical Center, Donders Institute for Brain, Cognition and Behaviour, Nijmegen, The Netherlands; Dr. Robert F. Coen, Mercer's Institute for Research on Ageing, St. James’s Hospital, Dublin, Ireland; Kate E. Devenney, School of Medicine, Dublin, Ireland; Dr. Emer M. Guinan, School of Medicine, Trinity College Dublin, Ireland; Dr. Damien Ferguson, Department of Neurology, St. James’s Hospital, Dublin, Ireland; Prof. Roy P.C. Kessels, Department of Medical Psychology, Radboud University Medical Center, Donders Institute for Brain, Cognition and Behaviour, Nijmegen, The Netherlands; Prof. Brian Lawlor, Connolly Norman Professor of Old Age Psychiatry, Trinity College Institute of Neuroscience, Dublin, Ireland; Prof. Romain Meeusen, Human Physiology Research Group, Vrije Universiteit Brussels, Belgium; Prof. Christian Montag, Institute of Psychology and Education, Ulm University, Germany and Key Laboratory for NeuroInformation / Center for Information in Medicine, and School of Life Science and Technology, University of Electronic Science and Technology of China, Chengdu, China; Dr Ross T. Murphy, St. James’s Hospital, Dublin, Ireland; Prof. Marcel G.M. Olde Rikkert, Department of Geriatric Medicine, Radboud Alzheimer Centre, Radboud University Medical Center, Nijmegen, The Netherlands; Prof. M. Cristina Polidori, Ageing Clinical Research, University Hospital of Cologne, Germany; Prof. Martin Reuter, University of Bonn, Department of Psychology and Center for Economics & Neuroscience, Laboratory of Neurogenetics, Bonn, Germany; Marit L. Sanders, Department of Geriatric Medicine, Radboud University Medical Center, Donders Institute for Brain, Cognition and Behaviour, Nijmegen, The Netherlands; Prof. Stefan Schneider, Institute of Movement and Neurosciences, German Sport University Cologne, Germany and Faculty for Science, Health, Education and Engineering, University of the Sunshine Coast, Australia; Prof. Heiko K. Strüder, Institute of Movement and Neurosciences, German Sport University Cologne, Germany; Tim Stuckenschneider, Institute of Movement and Neurosciences, German Sport University Cologne, Germany; Prof H.J. Thijssen, Department of Physiology, Radboud University Medical Center, Nijmegen, The Netherlands; Prof. Tobias Vogt, Institute for Professional Sport Education and Sport Qualifications, German Sport University Cologne, Germany; Prof. Cathal Walsh, University of Limerick, Ireland; Prof. Bernd Weber, Department of Epileptology, University Hospital Bonn, Germany.

We would like to acknowledge the following network of hospital sites and investigators who have assisted in recruiting participants to this study at the Dublin site; Dr Jennifer Hoblyn, Bloomfield Healthcare; Dr Andrew Eustace, Highfield Healthcare; Dr Cora McGreevy, Mater Misericordiae University Hospital; Dr Aisling Denihan from Old Age Psychiatry in Navan; Dr Justin Kinsella, St. Vincent’s University Hospital; Dr Declan Lyons, St. Patrick’s University Hospital and Dr Sean Kennelly, Tallaght Hospital.

It was also noticed after publication that the following errors occur in the Collaborators section on PubMed due to incorrect marking in the XML:

The name Classen JA is incorrect, but should be Claassen JA.

The author Stuckenschneider T is omitted from this list, but should appear between Strüder HK and Thijssen DH.

These authors appear correctly in the Acknowledgments section, listed as part of the NeuroExercise study group in all full text versions.

## References

[1] Devenney KE, Sanders ML, Lawlor B, Olde Rikkert MGM, Schneider S, the NeuroExercise study group (2017). The effects of an extensive exercise programme on the progression of mild cognitive impairment (MCI): study protocol for a randomised controlled trial. BMC Geriatr.

